# MicroRNA-101 inhibits invasion and angiogenesis through targeting ITGA3 and its systemic delivery inhibits lung metastasis in nasopharyngeal carcinoma

**DOI:** 10.1038/cddis.2016.486

**Published:** 2017-01-19

**Authors:** Xin-Ran Tang, Xin Wen, Qing-Mei He, Ying-Qin Li, Xian-Yue Ren, Xiao-Jing Yang, Jian Zhang, Ya-Qin Wang, Jun Ma, Na Liu

**Affiliations:** 1 Sun Yat-sen University Cancer Center, State Key Laboratory of Oncology in South China, Collaborative Innovation Center of Cancer Medicine, 651 Dongfeng Road East, Guangzhou, People's Republic of China

## Abstract

Clinically, distant metastasis after primary treatment remains a key problem in nasopharyngeal carcinoma (NPC), and the treatment outcome of metastatic NPC remains disappointing, so there is a pressing need to identify novel therapeutic strategies. In accordance with our previous microarray data, we found that miR-101 was downregulated in NPC clinical specimens and cell lines. Ectopic expression of miR-101 significantly suppressed NPC cell migration, invasion and angiogenesis *in vitro* and inhibited angiogenesis and metastasis *in vivo* using the chicken chorioallantoic membrane model. Furthermore, *ITGA3* was identified and validated as a novel target of miR-101, and the restoration of ITGA3 expression potently rescued the suppressive effects of miR-101. In addition, NPC patients with high ITGA3 expression had poorer overall survival and distant metastasis-free survival than patients with low ITGA3 expression, and ITGA3 overexpression was an independent poor prognostic factor in NPC. More importantly, we demonstrated that the systemic delivery of lentivirus-mediated miR-101 abrogated the lung metastatic colonization formation of NPC cells without obvious toxicity. Our study elucidates the molecular mechanisms of miR-101/ITGA3 pathway in regulating NPC metastasis and angiogenesis, and the systemic delivery of miR-101 provides a potent evidence for the development of a novel microRNA-targeting anticancer strategy for NPC patients.

Nasopharyngeal carcinoma (NPC) is a cancer arising from the nasopharynx epithelium. It is notable for its unique pattern of geographical distribution, with a high prevalence in South China, Southeast Asia, North Africa, the Middle East and Alaska.^1, 2, 3^ The majority of NPC patients are diagnosed with advanced stages at their first visit. Although the local and regional control of NPC patients have been significantly improved due to improved radiotherapy technologies and broader application of chemotherapy,^4, 5^ more than 20% of them eventually develop distant metastasis after treatment.^6^ Therefore, understanding the mechanisms underlying NPC metastasis is of crucial importance to identify novel therapeutic targets.

MicroRNAs (miRNAs) are a family of 21–25 nucleotide small non-coding RNA molecules that negatively regulate gene expression in a sequence-specific manner. Increasing evidence indicates that they potently influence cellular behaviour through the regulation of extensive gene expression networks.^7, 8^ High throughput techniques such as microarrays or next-generation sequencing show that miRNAs are dysregulated in almost all types of human cancer, including NPC. MiRNAs function as either tumour suppressors or oncogenes, exerting a variety of roles in cancer development and progression.^9, 10^ Therefore, the modulation of a single miRNA may affect several pathways simultaneously to achieve clinical benefit, and miRNAs have potential as therapeutic targets for the treatment of cancer.^10, 11^

Recently, it has been reported that several dysregulated miRNAs, such as miR-29c, miR-34c and miR-93, can regulate NPC cell invasion and metastasis.^12, 13, 14^ These findings suggest that the dysregulation of miRNAs is involved in NPC development and progression; therefore, more intensive investigation could better elucidate NPC metastatic mechanisms and provide therapeutic targets for NPC patients. Based on our previous microarray analysis, miR-101 was found to be downregulated in NPC tissue samples.^15^ In this study, we aim to further evaluate the biological function and molecular mechanism of miR-101 in NPC metastasis, as well as the therapeutic efficacy of miR-101 restoration therapy in NPC mouse model.

## Results

### MiR-101 is downregulated in NPC clinical specimens and cell lines

**Figure 1:**
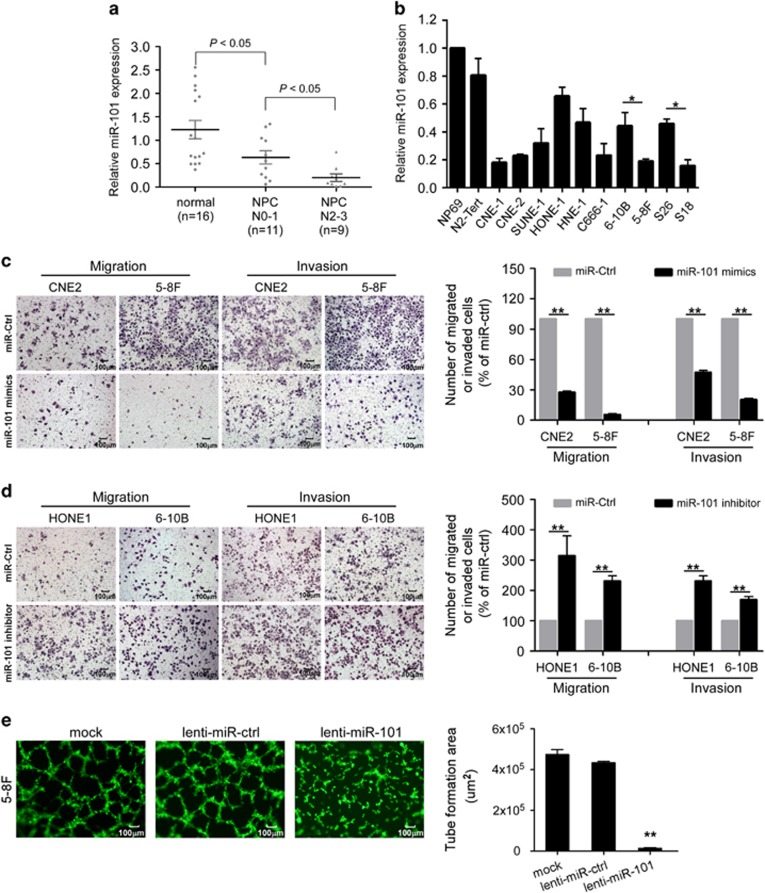
miR-101 is downregulated in NPC, and suppresses NPC cell migration, invasion and angiogenesis *in vitro.* (**a**) Relative expression of miR-101 in normal nasopharyngeal epithelial tissue samples (*n*=16), NPC tissues with low (*n*=11) or high (*n*=9) level regional lymph node metastasis. (**b**) Relative expression of miR-101 in immortalized nasopharyngeal epithelial cell lines NP69, N2-Tert and 10 NPC cell lines. U6 was used as an endogenous control. (**c**) Representative results of the Transwell migration and invasion assay for CNE-2 or 5–8F cells transfected with miR-101 mimic or miR-Ctrl. (**d**) Representative results of the Transwell migration and invasion assay for HONE1 or 6–10B cells transfected with miR-101 inhibitor or miR-Ctrl. (**e**) Representative results of vascular network formation of 5–8F cells overexpressing miR-101 or control and its quantification by measuring the tube formation area of the capillary-like structure. Data are presented as mean±S.D.; *P*-values were calculated using the Student's *t*-test, *P<0.05, **P<0.01

In accordance with our previous microarray data,^15^ quantitative RT-PCR confirmed that miR-101 was downregulated in 20 fresh-frozen NPC biopsy tissues compared with 16 normal nasopharyngeal epithelial tissues (Figure 1a, *P*<0.05). Moreover, miR-101 expression was much lower in primary NPC tissues with high level regional lymph node metastasis than those with low level regional lymph mode metastasis (Figure 1a, *P*<0.05). Furthermore, we evaluated the expression level of miR-101 in NPC cell lines, and confirmed that miR-101 was downregulated in all of the 10 tested NPC cell lines compared with two immortalized nasopharyngeal epithelial cell lines (Figure 1b). Furthermore, the expression of miR-101 was downregulated in cell lines with high metastatic potential (5–8F, S18) compared with cell lines with low metastatic potential (6–10B, S26) (Figure 1b; *P*<0.05). Taken together, these data suggest that miR-101 is downregulated in NPC and there is a possible link between reduced miR-101 expression and NPC metastasis.

### MiR-101 suppresses NPC cell migration, invasion and angiogenesis *in vitro*

To investigate the effect of miR-101 on NPC cell migration, invasion and angiogenesis, we performed Transwell migration and invasion assays and vascular mimicry assay. The Transwell migration and invasion assays demonstrated that the number of migrated or invaded cells were much smaller in CNE-2 and 5–8F cells transfected with miR-101 mimic than cells transfected with miR controls (Figure 1c, both *P*<0.01). However, depletion of endogenous miR-101 expression using miR-101 inhibitor markedly increased the migratory and invasive ability of HONE-1 and 6–10B cells (Figure 1d, both *P*<0.01). Next, vascular mimicry assay showed that 5–8F cells stably expressing lentivirus vector underwent a conspicuous rearrangement and formed a vasculogenic network within 22 h. In contrast, 5–8F cells stably overexpressing miR-101 showed a significant destruction of vascular mimicry structures and formed a significantly smaller total tube areas (Figure 1e, *P*<0.01). These results suggest that miR-101 can suppress the migratory, invasive and angiogenic ability of NPC cells *in vitro*.

### MiR-101 inhibits NPC cell angiogenesis and metastasis in a CAM model *in vivo*

**Figure 2:**
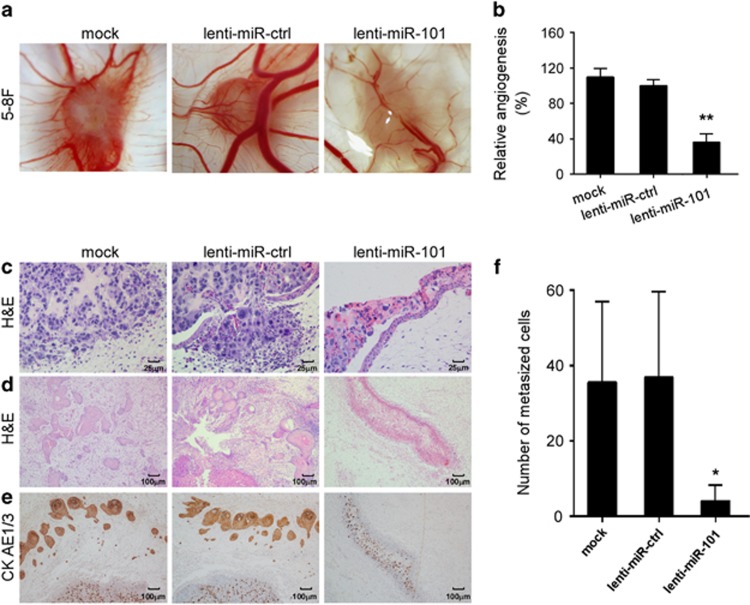
Restoring miR-101 expression suppresses NPC cell angiogenesis and metastasis *in vivo.* CAM angiogenesis and invasion assays were performed with 5–8F cells stably overexpressing miR-101 or control. (**a**) Representative images of new blood vessel formation. (**b**) Quantification of the average number of new blood vessels (*n*=6 for each group). (**c**) H&E staining of the local intravasation ( × 400). (**d**) H&E staining of the derived xenografts ( × 100). (**e**) IHC staining of CKAE1/AE3 ( × 100). (**f**) Number of metastatic 5–8F cells spread to the lung (*n*=6 for each group). Data are presented as mean ±S.D.; *P*-values were calculated using the Student's *t*-test, *P<0.05, **P<0.01

The *in vitro* results led us to examine the effect of miR-101 on NPC cell angiogenesis and metastasis *in vivo* using the chick chorioallantoic membrane (CAM) assay. As shown in Figure 2a, a markedly reduced number of blood vessels formed in the miR-101 stably overexpressing group compared with mock and lentivirus vector control groups, which was further validated by quantification analysis (Figure 2b, *P*<0.01). Moreover, hematoxylin and eosin (H&E) staining showed that control 5–8F cells that were implanted onto the CAM invaded into the connective tissues through the breached basement membrane, while cells stably overexpressing miR-101 failed to invade the basement membrane (Figure 2c). In addition, fewer tumour cell nests were present in the chorioallantoic mesenchymal in the miR-101 stably overexpressing group (Figure 2d), and immunohistochemistry staining of cytokeratin CKAE1/3 confirmed the epithelial origin of micro-tumours (Figure 2e). Taqman human Alu-specific PCR further validated that the number of cells metastasized to the lung with inoculated CAMs was much lower for miR-101 stably overexpressing group than controls (Figure 2f, *P*<0.05), which indicated a diminished intravasation and metastatic ability. The CAM data further indicate that miR-101 inhibits the ability of angiogenesis, intravasation and metastasis of NPC cells *in vivo*.

### ITGA3 is a functional target of miR-101 in NPC

**Figure 3:**
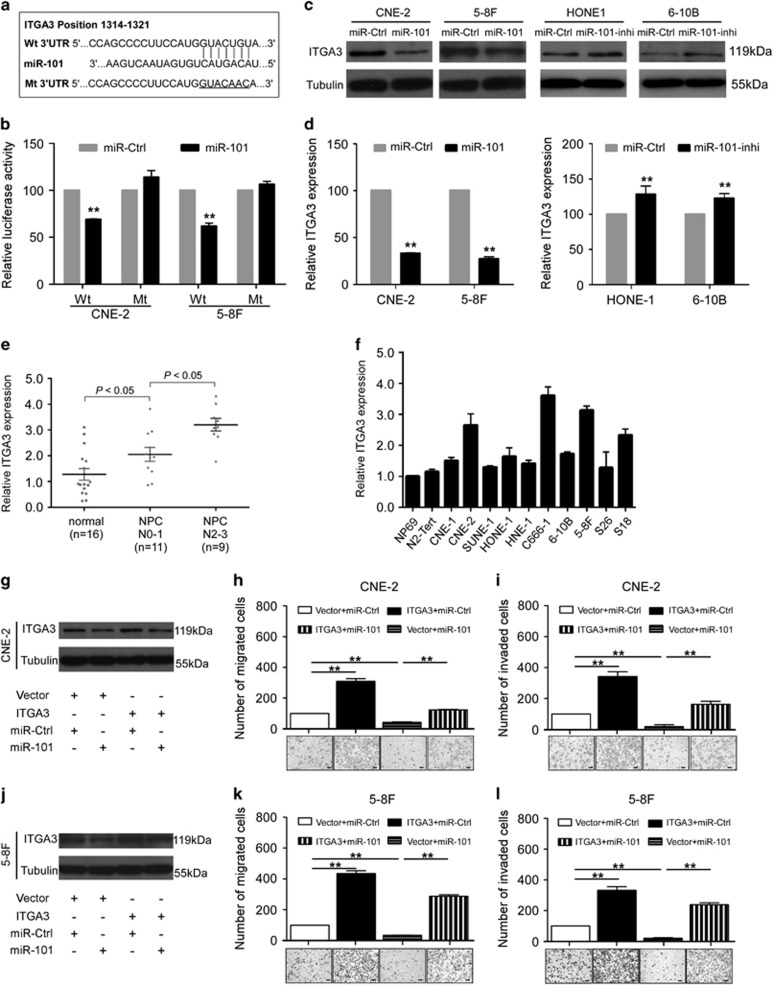
ITGA3 is a direct target of miR-101 and rescues its suppressive effects in NPC. (**a**) The putative binding sequence of miR-101 in ITGA3 3′-UTR; mutations were generated as indicated. (**b**) Relative luciferase activity of CNE-2 and 5–8F cells after co-transfected with wild-type or mutant ITGA3 3′-UTR reporter genes and miR-101 mimic or miR-ctrl. (**c**, **d**) Relative expression of ITGA3 mRNA and protein in CNE-2 and 5–8F cells transfected with miR-101 mimic or miR-ctrl, and in HONE1 and 6–10B cells transfected with miR-101 inhibitor or miR-ctrl. (**e**) Relative expression of ITGA3 in normal nasopharyngeal epithelial tissue samples (*n*=16), NPC tissues with low (*n*=11) or high (*n*=9) level regional lymph node metastasis. (**f**) Relative expression of miR-101 in immortalized nasopharyngeal epithelial cell lines NP69, N2-Tert and 10 NPC cell lines. (**g**–**i**) Effects of restoration of ITGA3 on migration and invasion in CNE-2 and 5–8F cells determined by Transwell migration and invasion assays. Data are presented as mean±S.D.; *P*-values were calculated using the Student's *t*-test, *P<0.05, **P<0.01

To explore the mechanism by which miR-101 inhibit NPC angiogenesis and metastasis, we identified integrin subunit alpha 3 (ITGA3) as a potential target of miR-101 by TargetScan and miRanda (Figure 3a). Luciferase reporter assay showed that the luciferase activity of the wild-type ITGA3 3′ UTR reporter gene was significantly reduced in CNE-2 and 5–8F cells transfected with miR-101 mimic, whereas the mutant reporter gene was not affected (Figure 3b, *P*<0.01), which confirmed that ITGA3 is a direct target of miR-101. Quantitative RT-PCR and western blotting showed that the ITGA3 expression was markedly decreased in miR-101-overexpressed NPC cells at both the mRNA and protein levels, while its expression was obviously increased in miR-101-knockdown cells, (Figures 3c and d). In addition, quantitative RT-PCR also showed that ITGA3 was upregulated in NPC tissue samples, especially tissues with high level regional lymph node metastasis, and in NPC cell lines (Figures 3e and f). Furthermore, CNE-2 and 5–8F cells were co-transfected with miR-101 mimic or miR-ctrl and either the empty vector or pEZ-Lv105-ITGA3 plasmid, which encoded the full-length coding sequence of ITGA3 without its 3′ UTR (Figures 3g and j). Opposite to the function of miR-101, ITGA3 significantly promoted NPC cell migration and invasion, and ectopic expression of ITGA3 abrogated the inhibitory effects on migration and invasion induced by miR-101 in NPC cells (Figures 3h, i, k and l, *P*<0.01). These results demonstrate that ITGA3 is a functional target of miR-101 in NPC.

### ITGA3 overexpression is associated with poor prognosis in NPC patients

**Figure 4:**
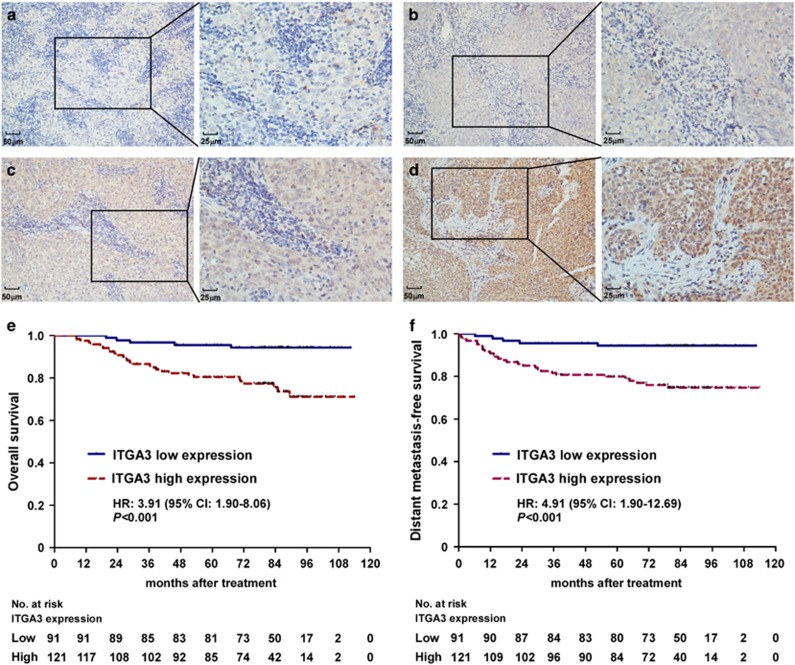
Overexpression of ITGA3 is associated with poor prognosis in NPC patients. Immunohistochemical detection of ITGA3 expression in 212 patients diagnosed with NPC. (**a**–**d**) Representative images of ITGA3 protein expression with negative staining (**a**), weak staining (light yellow, **b**), moderate staining (yellow brown, **c**) and strong staining (brown, **d**). All images are × 200 (left) and × 400 (right). (**e**, **f**), Kaplan–Meier analysis of overall survival (**e**) and distant metastasis-free survival (**f**) for NPC patients stratified by low and high expression of ITGA3. HR, hazard ratio; CI, confidence interval; HR values were calculated by unadjusted Cox regression analysis and *P*-values were calculated using unadjusted log-rank test

To further evaluate the clinical significance of ITGA3, the protein expression level of ITGA3 was determined in 212 paraffin-embedded NPC tissues using immunohistochemistry staining (Figures 4a–d). An optimal cutoff value (staining index: 8) for high and low ITGA3 expression was determined using ROC curve analysis,^16^ and 121 of the 212 (57.1%) samples were classified as high ITGA3 expressing (staining index≥8). As shown in Table 1, high ITGA3 expression was obviously associated with patients' sex, VCA-IgA, TNM-stage, distant metastasis and patients' death (all *P*<0.05). No significant correlations were found between ITGA3 expression and any other clinical features (Table 1). Further survival analysis established that NPC patients with high ITGA3 expression had significantly poorer overall survival (OS; HR, 3.91; 95% CI, 1.90–8.06; *P*<0.001) as well as distant metastasis-free survival (DMFS; HR, 4.91; 95% CI, 1.90–12.69; *P*<0.001). Furthermore, multivariate Cox regression analysis found that ITGA3 expression was an independent prognostic factor for OS (HR, 3.73; 95% CI, 1.80–7.72; *P*<0.001) and DMFS (HR, 4.65; 95% CI, 1.79–12.0; *P*=0.002). These results suggest that upregulation of ITGA3 is correlated with unfavourable clinical outcomes in NPC patients.

### Systemic delivery of miR-101 suppresses lung metastasis in NPC mouse model

**Figure 5:**
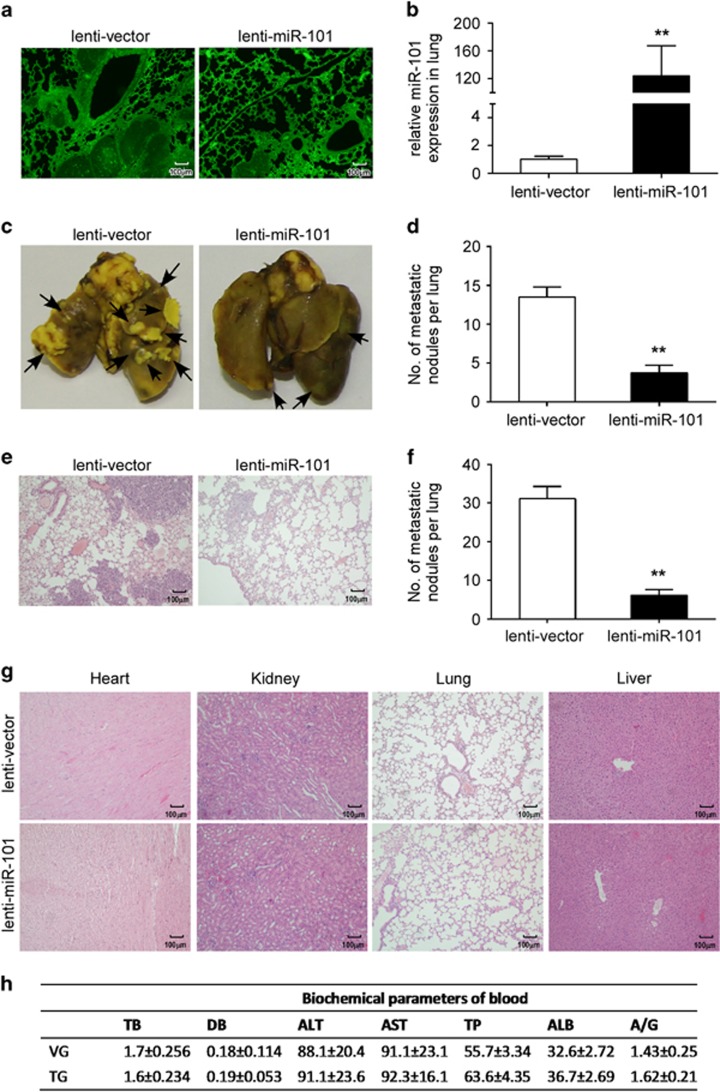
Systemic delivery of miR-101 suppresses lung metastasis in NPC mouse model. (**a**) Laser confocal microscopy showed efficient delivery of lentivirus to the lung, as indicated by coGFP expression. (**b**) The levels of miR-101 in the lung were significantly higher in lent-miR-101 treated mice than that in control mice (*n*=8 for each treatment group). (**c**, **d**) Representative images and quantification of macroscopic lung metastatic nodules, arrowheads indicate the metastatic nodules; (**e**, **f**) Representative images and quantification of microscopic lung metastatic nodules stained with H&E. (**g**) H&E staining of heart, kidney, lung and liver. (**h**) Main serological parameters of mice at the study endpoints. VG: vector group; TG: treatment group; TB: total bilirubin (μmol/l); DB: direct bilirubin (μmol/l); ALT: alanine aminotransferase (U/I); AST: aspartate aminotransferase (U/I); TP: total protein (g/l); ALB: albumin (g/l); A/G: albumin/globulin ratio. Data are presented as mean±S.D.; *P*-values were calculated using the Student's *t*-test, **P<0.01

Due to the effective role of miR-101 in the inhibition of NPC metastasis and angiogenesis, we then evaluated the therapeutic efficacy of miR-101 in a mouse model of lung metastasis. Two weeks after the preparation of the lung colonization model, lent-miR-ctrl or lent-miR-101 was, respectively, administered to mice via tail vein two times a week for a month. Two weeks later, mice were killed and tumour burden was assessed. As shown in Figure 5a, the levels of coGFP in the lung tissues of lent-miR-101 or lent-miR-ctrl treated mice were similar, exhibiting a high infectious efficiency, while the levels of miR-101 in the lung tissues of lent-miR-101 treated mice were significantly higher than control mice (Figure 5b, *P*<0.01). As for the treatment effect, the number of macroscopic and microscopic metastatic nodules formed in the lung tissues of lent-miR-101 mice group was remarkably smaller than the lent-miR-ctrl mice group (Figures 5c–f, both *P*<0.01). Notably, no acute liver toxicity and other organ toxicity were observed in mice after the delivery of lent-miR-101 or lent-miR-ctrl, which were determined by histological dissection (Figure 5g) and serological markers (Figure 5h). These results validate that the invasive behaviour of NPC could be suppressed by miR-101 administration.

## Discussion

Clinically, distant metastasis after primary treatment remains the key problem of NPC, and the treatment outcome of metastatic NPC remains disappointing. Although numerous mediators of metastasis have been identified in NPC, these factors are generally difficult to be targeted. Recent advancement in miRNA-based therapy has shown to be a more viable way for anti-cancer therapy and various delivery strategies have been developed.^11, 17, 18, 19^ A perfect example of miRNA replacement therapy is MRX34, a liposome-based miR-34 mimic who has became the first miRNA to enter phase I clinical trials in 2013 (http://clinicaltrials.gov/ct2/show/NCT01829971).^20^ Therefore, it is important to identify the critical miRNAs that are associated with the metastatic process of NPC, so that efficient therapeutic agents could be appropriately tested.

MiRNAs are small non-coding RNA molecules and function as master regulators of gene expression at the post-transcriptional level with a sequence-specific manner.^7, 8^ Increasing evidence indicates that miRNAs are dysregulated in most types of cancers and play a vital role in the processes of tumour invasion and metastasis.^21^ Recently, our microarray analysis demonstrated that miR-101 was downregulated in NPC. In the present study, we confirmed that miR-101 was frequently downregulated in NPC cell lines and freshly frozen clinical samples; the same deregulation has also been observed by other groups.^22^ Ectopic expression of miR-101 suppressed NPC cell migration, invasion and angiogenesis *in vitro*, while silencing of miR-101 promoted cell migration and invasion *in vitro*. Moreover, our CAM data strongly implicated that miR-101 was involved in regulating NPC tumour invasion and metastasis through its activity in tumour cell intravasation, extravasation and subsequent cell seeding.

MiRNAs exert their function through base-paring to the 3′ UTR of their target gene with imperfect complementarity.^7^ MiR-101 has many validated oncogenic targets, including FOS, EZH2, MCL-1 and ROCK2.^23, 24, 25, 26^ It has been reported that miR-101 could inhibit migration and invasion by targeting AP-1 pathway in hepatoma cells,^27^ and a more recent study shows that loss of miR-101 positively correlates with the upregulation of COUP-TFII activity, which contributes to prostate cancer metastasis.^28^ These data suggest a powerful tumour suppressive activity of miR-101 in human cancers. In our present study, we identified ITGA3 as a potential target of miR-101 using two open-access databases. Next, using luciferase reporter assays, we validated ITGA3 as a novel target of miR-101. We observed that overexpression of miR-101 markedly reduced ITGA3 mRNA and protein expression, and the restoration of ITGA3 rescued the suppressive effects of miR-101 on NPC migration and invasion.

Based on the demonstration of the function and mechanism of miR-101 involved in NPC metastasis, we further tested the hypothesis that miR-101 could serve as a therapeutic target for NPC In this study, we chose a lentivirus-based system as miR-101 administration vehicle, since its safety has been tested in preclinical research and clinical trials and it could provide a therapeutic benefit for the patients.^29, 30^ In our mouse NPC lung metastatic model, we did observe that the systemic delivery of lent-miR-101 exhibited a high infectious efficiency and a high expression level of miR-101 in the lungs of mice without obvious toxicity, demonstrating lentivirus as an effective and non-toxic means to deliver miRNAs to mouse. More importantly, systemic delivery of lent-miR-101 suppressed the formation of metastatic nodules in lungs of mice. Overall, our data hereby provide a basis for the concept that the systemic administration of miR-101 mediated by lentivirus might be a clinically viable anti-NPC therapeutic strategy.

In conclusion, we demonstrate that miR-101 is downregulated in NPC, and can suppress NPC angiogenesis and metastasis *in vitro* and *in vivo* by targeting ITGA3. More importantly, we demonstrated that systemic administration of lentivirus-mediated miR-101 abrogated NPC lung metastatic ability in a mouse model without obvious toxicity, which provides a potent evidence for the development of a novel miRNA-targeting anticancer therapy.

## Materials and methods

### Clinical specimens

Twenty freshly frozen NPC biopsy samples and 16 normal nasopharyngeal epithelia specimens were collected from Sun Yat-sen University Cancer Center. Besides, 212 paraffin-embedded NPC tissue samples with detailed clinical characteristics and long-term follow-up data, which had been collected from 2006 to 2008, were obtained from the same cancer centre. None of the patients had received any anti-tumour therapy before biopsy. The research protocols were approved by the Institutional Ethical Review Board of Sun Yat-sen University Cancer Center, and informed consents have been obtained from all patients.

### Cell lines

Eight NPC cell lines (CNE-1, CNE-2, C666-1, HNE-1, HONE-1, SUNE-1, 5–8F, 6–10B) were cultured in RPMI-1640 (Invitrogen, Carlsbad, CA, USA) supplemented with 10% FBS (Gibco, Grand Island, NY, USA), and the other two cell lines (S18 and S26) were cultured in DMEM (Invitrogen) supplemented with 10% FBS. The human immortalized nasopharyngeal epithelial cell lines (NP69, NPEC-tert) were cultured in keratinocyte/serum-free medium (Invitrogen) supplemented with bovine pituitary extract (BD Bioscience, San Diego, CA, USA). All the immortalized nasopharyngeal epithelial cell lines and NPC cell lines that had been authenticated were generously provided by Dr M. Zeng (Sun Yat-sen University Cancer Center). 293FT cells from ATCC were cultured in DMEM supplemented with 10% FBS.

### RNA extraction and quantitative RT-PCR

Total RNA was extracted using TRIzol reagent (Invitrogen) and reverse-transcribed using M-MLV reverse transcriptase (Promega, Madison, WI, USA) with Bulge-Loop miRNA-specific RT primers (RiboBio, Guangzhou, China) for miR-101 or random primers (Promega) for ITGA3. Quantitative PCR reactions were conducted on the Bio-Rad CFX96 Touch sequence detection system (Bio-Rad Laboratories Inc, Hercules, CA, USA) using Platinum SYBR Green qPCR SuperMix-UDG reagents (Invitrogen). All reactions were done in triplicate, reactions with no template or reverse transcriptase were used as negative controls. U6 or GAPDH were used as endogenous controls for miR-101 and ITGA3, respectively, and the relative expression was calculated with the 2^−ΔΔCT^ equation.

### Oligonucleotide and plasmid transfection

miR-101 mimic, inhibitor and their corresponding controls (GeneParma, Suzhou, China) were transfected into NPC cells using Lipofectamine 2000 reagent (Invitrogen). Cells were also co-transfected with miR-101 mimic or miR-Ctrl and either the empty vector control (Vector) or pEZ-Lv105-ITGA3 (ITGA3) plasmid overexpressing ITGA3 (FulenGen, Guangzhou, China) using Lipofectamine 2000 reagent. Cells were harvested for assays 48 h after transfection.

### Generation of stably transfected cell lines

The pri-miR-101 sequence was cloned into the lentiviral plasmid pSin-EF2-puromycin (Addgene, Cambridge, MA, USA); pSin-EF2-miR-101-coGFP or negative control pSin-EF2- coGFP vector was then co-transfected into 293FT cells with the psPAX2 packaging plasmid (Addgene) and the pMD2.G envelope plasmid (Addgene) using the calcium phosphate method.^31^ After transfection, the cell supernatants were harvested and used to infect 5–8F cells, and stably transfected cells were selected using puromycin and validated by quantitative RT-PCR.

### Migration and invasion assays

For the Transwell migration and invasion assay, transfected cells were harvested and suspended in serum-free medium, and 5 × 10^4^ or 1 × 10^5^ cells were seeded onto the upper 8-μm pore size Transwell chambers (Corning, NY, USA) coated without or with Matrigel (BD Biosciences, San Diego, CA, USA), and media supplemented with 10% FBS was placed into the lower chamber as a chemoattractant. After 12 or 24 h, the cells that had migrated or invaded into the lower surface were fixed, stained and photographed with an inverted microscope (Olympus, Tokyo, Japan).

### Capillary-like structure formation assay: vascular mimicry

Capillary-like structure formation assay was performed as previously described.^32^ Briefly, 2 × 10^4^ transfected 5–8F (GFP) cells were seeded onto Matrigel (BD Biosciences) in 96-well plates. After 22-h incubation at 37 °C, the capillary-like tubes were analysed using an inverted light microscope at × 100 magnification. The corresponding areas were analysed using the NIH Image J software package.^33^

### CAM angiogenesis and invasion assays

CAM assays were performed as previously described.^34, 35^ Fertilized chicken eggs (Guangdong Academy of Agricultural sciences, Guangzhou, China) were incubated at 38 °C in 70% humidity for 7 days. Fifteen microliters of 5-8F cells (2 × 10^6^ cells) in mixture with 15 μl Matrigel (BD Biosciences) was implanted in each egg and then returned to the incubator; six viable embryos were tested for each assay per group. For angiogenesis assays, the area around the implanted Matrigel was photographed 72 h after the implantation and calculated for branching of blood vessels.^36^ For local invasion assay, CAMs were isolated at 72 h. Invading cells were then examined by HE staining and human cytokeratin (CK AE1/AE3, ZM-0069, ZSGB-BIO, Beijing, China). For metastasis assays, embryonic lungs were isolated on day 18 of embryonic growth. Genomic DNA were prepared using the Gentra Puregene Tissue Kit (158667, Qiagen) and then analysed for tumour cells by Taqman human Alu-specific PCR.^34^

### Western blot analysis

Total protein was separated on 8% SDS-PAGE gels and transferred onto polyvinylidene fluoride membranes (Merck Millipore, Billerica, MA, USA). The membrane was blocked with 5% non-fat milk and incubated with rabbit anti-ITGA3 antibody (Abcam, Cambridge, UK, 1:1000 dilution) and mouse α-tubulin antibody (1:1000; Sigma-Aldrich, Ronkonkoma, NY, USA) was used as a loading control. The proteins were detected by enhanced chemiluminescence reagents.

### Luciferase report assay

The ITGA3 Wt and Mt 3′ UTR were cloned into the Xhol and Notl restriction sites of the psiCHECK-2 luciferase reporter plasmid (Promega). For the luciferase assay, cells were seeded into six-well plates for 24 h, and then co-transfected with the ITGA3 Wt or Mt 3′ UTR reporter plasmids, and miR-Ctrl or miR-101 mimic using Lipofectamine 2000 reagent (Invitrogen), as well as the control vector pRL-TK (Promega). Luciferase activities were determined using the Dual-Luciferase Reporter Assay System (Promega).

### Immunohistochemistry

5-*μ*m tissue sections were cut, deparaffinized, rehydrated and subjected to high-pressure for antigen retrieval in EDTA antigen retrieval buffer. 3% hydrogen peroxide was used to inactivate endogenous peroxidase activity, and non-specific binding was blocked using 1% bovine serum albumin. The sections were incubated overnight at 4 °C with rabbit monoclonal anti-ITGA3 antibody (1 : 750; Abcam); normal goat serum was used as a negative control. The subsequent steps were performed using the Daco REAL EnVision Detection system (DACO, Denmark). The sections were scored independently by two pathologists, and the staining index was generated as the product of the staining intensity (0, no staining; 1, weak, light yellow; 2, moderate, yellow brown; 3, strong, brown) and the proportion of positive cells (1, <10% 2, 10–35% 3, 35–70%, 4, >70%).^37^

### Systemic administration of lenti-miR-101 to a lung metastasis mouse model

Male BALB/c nude mice aged 4–6 weeks were purchased from the Medical Experimental Animal Center of Guangdong Province (Guangzhou, China). To establish a lung metastasis mouse model, 1 × 10^6^ 5–8F cells suspended in 200 μl PBS were injected via the tail vein. After 2 weeks, lentivirus-miR-101-coGFP (lent-miR-101), or lentivirus-miR-ctrl-coGFP (lent-miR-ctrl) were injected via tail vein at a dose of 10^8^ MOI per mouse (200 μl) using a 30-gauge ultra-fine insulin syringe two times a week for a month. Two weeks later, the mice were euthanized using cervical dislocation method, the lung tissues were fixed, paraffin-embedded and consecutive 5-μm sections were made for each block of the lung and one every 10 sections were stained with H&E staining. The number of macroscopic and microscopic metastatic nodules in the lungs were counted. All animal research protocols were approved by the Institutional Animal Care and Use Ethics Committee of our cancer center.

### Statistical analysis

Statistical analysis for data included the χ^2^ test, Fisher's exact test and Student's *t*-test. Survival curves were plotted by the Kaplan–Meier method and compared by the log-rank test. The significance of various variables for survival was analysed by univariate and multivariate Cox regression analyses. Data are presented as mean±S.D. SPSS 22.0 software (IBM, Armonk, NY, USA) was used for statistical analysis. All tests were two-tailed; *P*-values<0.05 were considered statistically significant.

## Figures and Tables

**Table 1:** Clinical characteristics of the patients with nasopharyngeal carcinoma stratified by low and high expression of ITGA3

**Characteristics**	**No. of patients**	**Expression of ITGA3**		***P***-**value**
		**Low** **(*****n*****=91)**	**High** **(*****n*****=121)**	
*Age (years)*				0.872
≤45	109	46	63	
>45	103	45	58	
				
*Sex*				**0.045**
Male	159	62	97	
Female	53	29	24	
				
*WHO type*				0.680
I+II	8	4	4	
III	204	87	117	
				
*VCA-IgA*				**0.017**
<1 : 80	23	15	8	
≥1 : 80	186	73	113	
				
*EA-IgA*				0.241
<1 : 10	42	21	21	
≥1 : 10	165	66	99	
				
*TNM stage*				**0.027**
I–II	58	32	26	
III–IV	154	59	95	
				
*Distant metastasis*				**<0.001**
Yes	34	5	29	
No	178	86	92	
				
*Death*				**<0.001**
Yes	49	9	40	
No	163	82	81	

Abbreviations: EA-IgA: early antigen immunoglobulin A; VCA-IgA: viral capsid antigen immunoglobulin A All patients were restaged according to the 7th edition of the AJCC Cancer Staging Manual. *P*-values were calculated using the chi-square test or Fisher's exact test, and significance was defined as *P* values of less than 0.05
